# Inclusion of Neuropsychological Scores in Atrophy Models Improves Diagnostic Classification of Alzheimer's Disease and Mild Cognitive Impairment

**DOI:** 10.1155/2015/865265

**Published:** 2015-05-25

**Authors:** Mohammed Goryawala, Qi Zhou, Warren Barker, David A. Loewenstein, Ranjan Duara, Malek Adjouadi

**Affiliations:** ^1^Center for Advanced Technology and Education, Department of Electrical and Computer Engineering, Florida International University, Miami, FL, USA; ^2^Wien Center for Alzheimer's Disease and Memory Disorders, Mount Sinai Medical Center, Miami Beach, FL, USA; ^3^Department of Psychiatry, Miller School of Medicine, University of Miami, Miami, FL, USA; ^4^Department of Neurology, Miller School of Medicine, University of Miami, Miami, FL, USA; ^5^Herbert Wertheim College of Medicine, Florida International University, Miami, FL, USA

## Abstract

Brain atrophy in mild cognitive impairment (MCI) and Alzheimer's disease (AD) are difficult to demarcate to assess the progression of AD. This study presents a statistical framework on the basis of MRI volumes and neuropsychological scores. A feature selection technique using backward stepwise linear regression together with linear discriminant analysis is designed to classify cognitive normal (CN) subjects, early MCI (EMCI), late MCI (LMCI), and AD subjects in an exhaustive two-group classification process. Results show a dominance of the neuropsychological parameters like MMSE and RAVLT. Cortical volumetric measures of the temporal, parietal, and cingulate regions are found to be significant classification factors. Moreover, an asymmetrical distribution of the volumetric measures across hemispheres is seen for CN versus EMCI and EMCI versus AD, showing dominance of the right hemisphere; whereas CN versus LMCI and EMCI versus LMCI show dominance of the left hemisphere. A 2-fold cross-validation showed an average accuracy of 93.9%, 90.8%, and 94.5%, for the CN versus AD, CN versus LMCI, and EMCI versus AD, respectively. The accuracy for groups that are difficult to differentiate like EMCI versus LMCI was 73.6%. With the inclusion of the neuropsychological scores, a significant improvement (24.59%) was obtained over using MRI measures alone.

## 1. Introduction

Perhaps one of the most challenging research issues in Alzheimer's disease (AD) is in identifying relevant measures which could define the different stages of AD as a progressive neurodegenerative disorder [1, 2]. Targeted treatment and early intervention procedures could be prescribed on the basis of such findings.

Brain imaging and neuropsychological testing are the main research domains used to determine specific cognitive, structural, functional, and biological measures to study AD and its prodromal stages. Structural MRI [3–7] and functional imaging modalities like Single-Photon Emission Computed Tomography (SPECT) [8, 9], Positron Emission Tomography (PET) [10, 11], synchronous neural interactions (SNI) obtained using magnetoencephalography (MEG) [12, 13], and Central Spinal Fluid (CSF) [6] as well as electroencephalography (EEG) [14–16] have been used with varying degrees of success in identifying AD. Clinicians regularly use these biomarkers as guides, and, more recently, combinations of two or more biomarkers are being explored to improve our understanding of AD [4–7, 10]. Exemplifying such combinations, biomarkers of MRI and CSF reportedly yield better accuracy as compared to their individual results. In similar studies, Fan et al. combined MRI and PET biomarkers [5], while the group of Walhovd et al. and the group of Zhang et al. worked on a combination of MRI, PET, and CSF biomarkers and reported results with conclusive indicators in the diagnosis of AD or Mild Cognitive Impairment (MCI) [4, 10].

Many other studies focused on the combination of neuropsychological testing with medical imaging modalities. In a notable study, Ewers and his colleagues combined the main biomarkers of MRI and CSF with neuropsychological tests to predict the conversion from MCI to AD [17]. Their study, which included 81 AD patients and 101 elderly control subjects, demonstrated that single-predictor models do yield comparable accuracies as multipredictor models. It showed that when the entorhinal cortex is used as the single predictor, the accuracy of the results ranged from the mid-60s to a high of 68.5%. In another study involving the prediction of MCI to AD conversion over a 2-year period, Gomar et al. researched the usefulness of combining different variables drawn from a series of biomarkers including cognitive markers and the different risk factors involved [18]. Using brain volumes, CSF and other cognitive markers, they determined that cognitive markers at baseline yield better predictors in the conversion of MCI to AD as compared to temporal neurobiological markers. They also show that, in contrast to biomarkers, a sharp decline in functional ability could serve as a better predictor in the conversion of MCI to AD. This latter finding concurs with their results that show that, with the inclusion of neuropsychological data, the accuracy increased to 90% in delineating AD patients from controls.

Both these studies, which primarily focus on the conversion process of MCI to AD, use a manual selection of the volumetric measures of the different regions of the brain and rely on the ADNI (Alzheimer's Disease Neuroimaging Initiative) public database. The proposed study, which relates well to these two studies, uses instead a fully automated approach to rank the neurobiological variables and volumetric measures. Thus, a more global approach is provided for constructing patterns of structural and physiological abnormalities in their entirety [5], with statistical proofs in support of the choice of the different variables and measures considered.

Other studies have focused their research efforts on determining the distinctive features that could delineate early MCI (EMCI) from late MCI (LMCI) [19, 20]. For example, Ferman et al. showed that nonamnestic MCIs (naMCI) were more likely to develop dementia with Lewy bodies (DLB), whereas patients with amnestic MCIs (aMCI) are more likely to convert to AD [21].

The proposed study examines the classification of AD, EMCI, and LMCI on the basis of a combination of subcortical and cortical MRI volumes with a slate of neuropsychological tests that include Mini-Mental State Examination (MMSE), Rey Auditory Verbal Learning Test (RAVLT), and Clinical Dementia Rating Scale Sum of Boxes Scores (CDRSb). The study reveals the importance of including neuropsychological tests in classifying the different stages of AD by using a combination of MMSE, RAVLT, and select volumetric variables. This study proposes also a fully automated feature extraction technique, with a ranking that provides statistical significance to the variables to be used in a multidimensional decisional space for optimal classification.

## 2. Methodology

**Figure 1:**
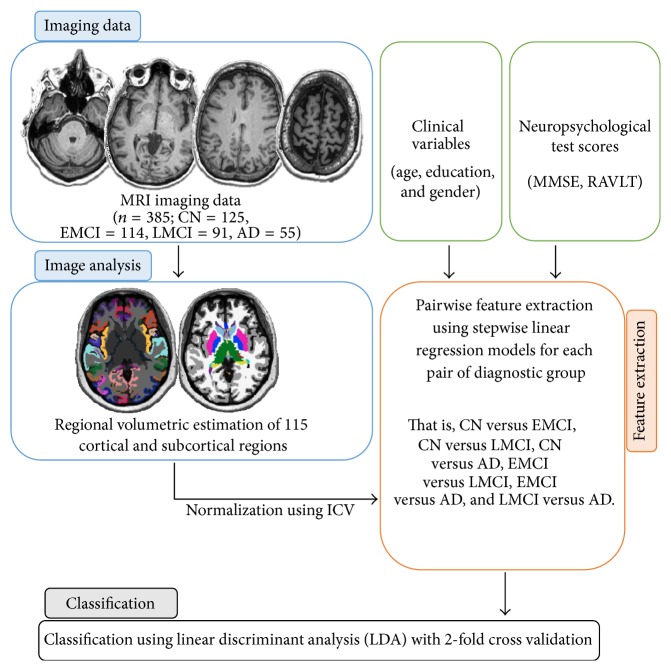
General construct of the classification study showing the main blocks, namely, image analysis, feature extraction, and classification.

Figure 1 illustrates the general structure of the entire process. The steps include acquisition of the MRI and neuropsychological parameters, the selection of significant variables using pairwise backward stepwise linear regression models, and the classification process using a well-established linear discriminant analysis (LDA). The fully automated data-driven technique allows for the possibility of replacing LDA with other algorithms such as Support Vector Machines (SVM) and Artificial Neural Networks (ANN) and probabilistic classifiers such as Quadratic Discriminant Analysis (QDA).

### 2.1. Study Data

Data used in this study were obtained from the ADNI database (http://adni.loni.usc.edu/). ADNI launched in 2003 aims to test whether magnetic resonance imaging, PET, other biological markers, and clinical and neuropsychological assessment can be combined to measure the progression of MCI and early AD.

Baseline demographic, clinical, neuropsychological, and volumetric MRI data for 385 subjects (55 diagnosed with mild AD and 91 with LMCI and 114 EMCI and 125 cognitively normal (CN) cases) were explored as outlined in Table 1.

All subjects had (1) a neurological and medical evaluation by a physician; (2) a full battery of neuropsychological tests [22], all in accordance with the National Alzheimer's Coordinating Center protocol (http://www.alz.washington.edu/), along with RAVLT [23]; and (3) structural volumetric MRI scans of the brain. The CDRSb was used as the index of functional ability, and the MMSE was used as the index of cognitive ability.

The cognitive diagnosis was made using a combination of the physician's diagnosis and neuropsychological diagnosis, as described previously [24]. The etiological diagnosis was made by the examining physician. The diagnosis of CN required that the physician's diagnosis was CN and no cognitive test scores were ≥1.5 SD below age- and education-corrected means. A probable AD diagnosis required a dementia syndrome and National Institute of Neurological and Communicative Disorders and Stroke/Alzheimer's Disease and Related Disorders Association criteria for AD [25].

### 2.2. Imaging Protocol

MRI scans were acquired on a 1.5-T machine (Siemen's Symphony, Iselin, NJ, USA, or General Electric, HDX, Milwaukee, WI, USA) using a proprietary 3D-magnetization-prepared rapid-acquisition gradient echo (3D MPRAGE) or 3D spoiled gradient echo sequences (FSPGR). Specifications for 3D MPRAGE include coronal sections with a 1.5 mm gap in thickness; section interval: 0.75 mm; TR: 2190 ms; TE: 4.38 ms; TI: 1100 ms; FA: 15°; NEX: 1; matrix: 256 × 256; FOV: 260 mm; bandwidth: 130 Hz/pixel; acquisition time: 9 minutes; and phase-encoding direction: right to left. Specifications for 3D FSPGR were the following: 140 contiguous coronal sections of 1.2 mm thickness; contiguous images with no section interval; TR; 7.8 ms; TE: 3.0 ms; inversion recovery preparation time: 450 ms; flip angle; 12°; NEX: 1; matrix: 256 × 256; FOV: 240 mm; bandwidth: 31.25 Hz/pixel; acquisition time: 6-7 minutes; and again phase-encoding direction: right to left.

### 2.3. Volumetric Correction

FreeSurfer pipeline (version 5.1.0) was applied to the MRI scans to produce 115 cortical and subcortical volumetric variables. These 115 regional volumes were corrected for head size variation using FreeSurfer's estimate of total intracranial volume (TIV), which has been considered highly accurate in adults [26]. Regional MRI volumes, normalized to total intracranial volume, were obtained from the ADNI database using data derived by researchers at the University of California, San Francisco, as part of the ADNI2 cohort. Each regional volume of the brain was divided by the subject's TIV to estimate regional volumetric ratios, which were used as features for the classification models. The volumes were not corrected for age, gender, and education since these demographic variables are later used as model terms in the statistical testing as detailed in Section 2.4.

### 2.4. Statistical Feature Extraction

Stepwise linear regression models (SLRM) have been widely used in studies related to Alzheimer's disease for assessing hippocampal atrophy [27], functional decline in cognitive ageing [28, 29], and cortical atrophy [30], to name a few.

**Figure 2:**
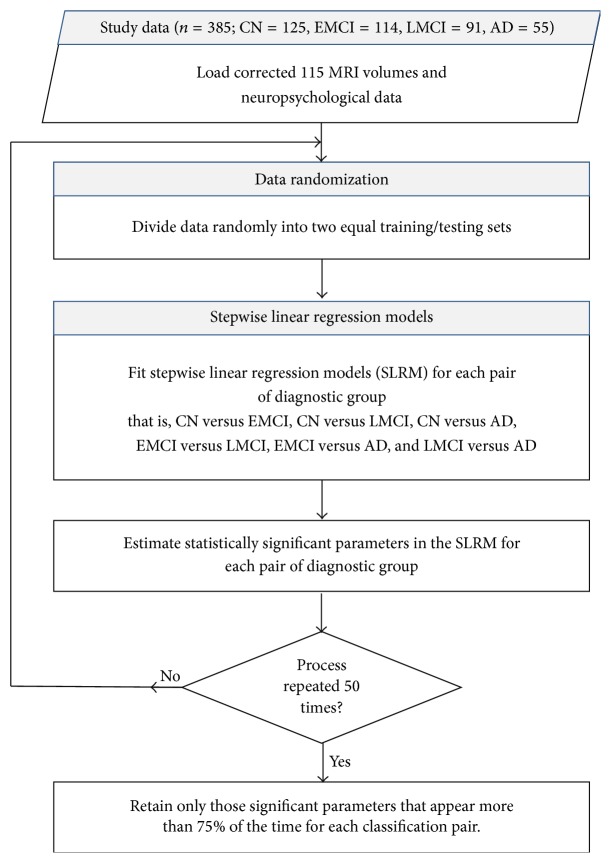
Procedural steps of the feature extraction technique using SLRM.

In this study, the SLRM method is used as a feature extraction technique to determine those variables (demographic, clinical, neuropsychological, or volumetric) that are significant towards classification of the different subtypes of the disease. The procedure followed is as shown in Figure 2.

The feature extraction step begins by randomly dividing the feature set of 120 parameters (age, gender, education, 2 neuropsychological test scores, namely, RAVLT and MMSE, and 115 corrected volumetric MRI regional ratios) into two groups. One of these groups is assigned as a training group and the other is discarded in this initial step. For the training group pairwise stepwise linear regression models are used to estimate the best set of parameters, which yields the highest correlation between the diagnostic gold standard and the feature set as given in (1). The pairwise models are trained for each of the six classification pairs, namely, CN versus EMCI, CN versus LMCI, CN versus AD, EMCI versus LMCI, EMCI versus AD, and LMCI versus AD. Consider

(1) $$\begin{array}{c} y=C_{0}+C_{1}*F_{1}+C_{2}*F_{2}+\cdots +C_{m}*F_{m}, \end{array}$$

where *y* is the model response (a logical variable showing the class in the pairwise classification pair), *F*_1_, *F*_2_,…, *F*_*m*_ are the *m* feature variables; *C*_1_, *C*_2_,…, *C*_*m*_ are their respective coefficients, and *C*_0_ is a constant. For the current study *m* = 121; however, the model is completely scalable to accommodate for fewer or larger number of features if needed.

The significance threshold for adding a feature to the model is fixed at 0.1 for the model* R* ^2^; that is, if the increase in the* R*-squared of the model is larger than 0.1, the corresponding feature is added to the model. On the other hand, a feature is removed from the model if the feature fails to improve the* R* ^2^ of the model by a number greater than 0.05. The choice of significant thresholds for adding or removing a term from the model was empirically adjusted so as to obtain a stable model, which can explain the variance in the data. Stepwise regression models, as executed in the paper, fit an initial model comprised of a single feature and then grow to accommodate other features. The choice of an increment in* R* ^2^ of 0.1 for adding a parameter to a model was to achieve a more conservative approach towards limiting feature space. Complex models with a large number of features often tend to overfit the model capturing the noise in the data rather than the underlying phenomenon. The removal threshold was fixed to 0.05 again to eliminate only weak features that resulted in lesser than 5% improvement in the model.

To account for the varied nature of the disease and the random distribution of the data under investigation, the SLRM are repeated 50 times for each diagnostic pair and only those features which appear to be significant more than 75% of the time are retained in the final feature set as was shown in Figure 2. The final features that are deemed significant are bound to constitute an optimal decisional space.

### 2.5. Linear Discriminant Analysis (LDA) Classifier

LDA is a technique widely used in pattern recognition, statistics, and machine learning, among others, for determining characteristic features that can aid in difficult segmentation tasks [31–33]. The LDA classifier used in this study attempts to estimate a posterior probability for each subject to enable its classification into either of the two groups for each of the six pairwise classifications.

The significant features determined in the feature extraction step are used to train a classifier to estimate the parameters of the linear discriminant functions for the two classes as given in

(2) d^1LF1,F2,…,Fm′=α1+α2∗F1+α3∗F2+⋯+αm′+1∗Fm′,d^2LF1,F2,…,Fm′=β1+β2∗F1+β3∗F2+⋯+βm′+1∗Fm′,

where *α*_1_, *α*_2_,…, *α*_*m*′_ and *β*_1_, *β*_2_, …, *β*_*m*′_ are the LDA parameters for the two groups, respectively, and *F*_1_, *F*_2_, …, *F*_*m*′_ are the set of significant parameters for each classification pair, where *m*′ ≤ *m*.

The training algorithm assumes a prior probability *p*_prior_ = 0.5, suggesting that a given subject has an equal probability of belonging to either one of the two classes. The classification algorithm assigns posterior probabilities *p*_1_ or *p*_2_ on the basis of the linear score *L*_1_ and *L*_2_, respectively, as described in (3). The posterior probabilities as calculated in (4) signify the likelihood of a subject to belong to either one of the groups. A higher posterior probability determines the grouping of the subjects:

(3) L1F1,F2,…,Fm′=d^1LF1,F2,…,Fm′+log⁡pprior,L2F1,F2,…,Fm′=d^2LF1,F2,…,Fm′+log⁡pprior

(4) p1=eL1F1,F2,…,Fm′eL1F1,F2,…,Fm′+eL2F1,F2,…,Fm′,p2=eL2F1,F2,…,Fm′eL1F1,F2,…,Fm′+eL2F1,F2,…,Fm′.

All of the experiments conducted in this study were based on 2-fold cross validation, distributing the subjects equally between training and testing sets. The training and testing sets were randomly assigned while the number of subjects in each group remained fixed. To limit the potential data portioning error introduced by random data assignment and cross validation, the same experiment with random data assignment was run 20 times and the average metrics of accuracy, sensitivity, specificity, precision, and* F*-measure are reported.

## 3. Results and Discussion

### 3.1. Significant Features

The significant features as determined by the SLRM technique for each of the six classifications are provided in Table 2. The results show that a combination, which includes neuropsychological parameters, demographic variables, and the volumetric variables, could act as the best linear model to estimate diagnostic patterns in pairwise comparisons. The number of features that are selected for each pairwise comparison varies from 5 in the case of LMCI versus AD to as many as 14 in the case of CN versus EMCI. On average, for diagnostic groups which are closely related to each other in disease progression, that is, CN versus EMCI, EMCI versus LMCI, and LMCI versus AD, a fewer number of significant parameters are seen as compared to groups that are diagnostically well separable. Such a trend was expected since closely related diagnostic groups have fewer marked atrophy changes that are visible with disease progression.

**Figure 3:**
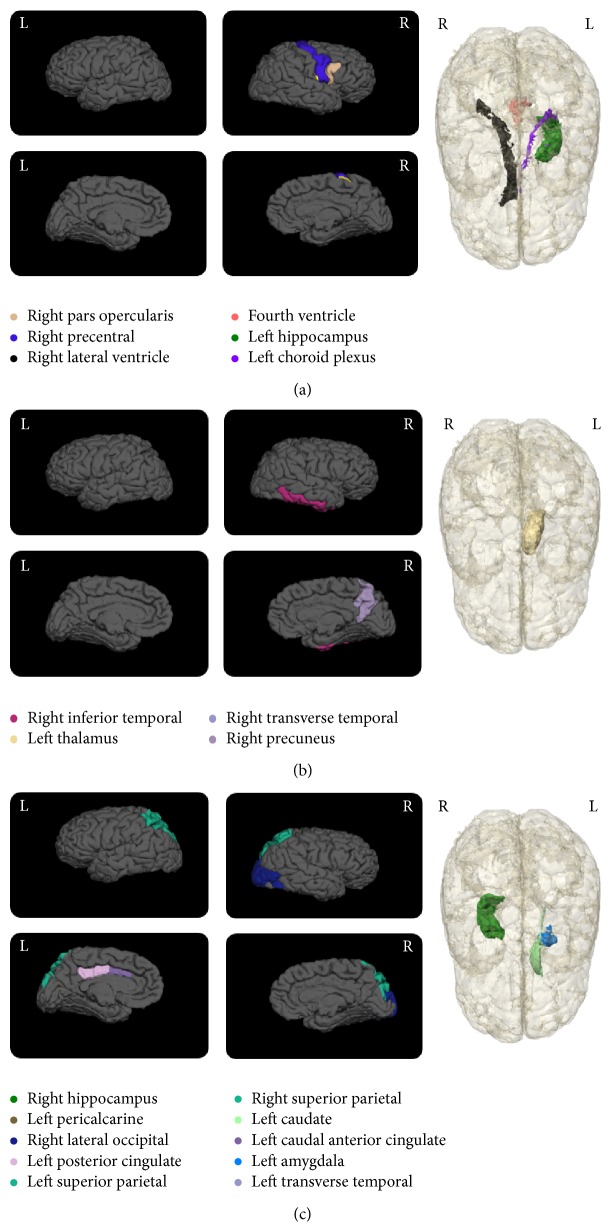
Cortical and subcortical maps showing the significant volumetric regions for each classification pair. (a) CN versus AD, (b) CN versus EMCI, and (c) CN versus LMCI. The regions are listed in Table 2. For each classification pair the letter “L” or “R” signifies the left and right hemisphere, respectively. Additionally, for the cortical representations, the top images in each set show the lateral view and the bottom images show the medial views.

**Figure 4:**
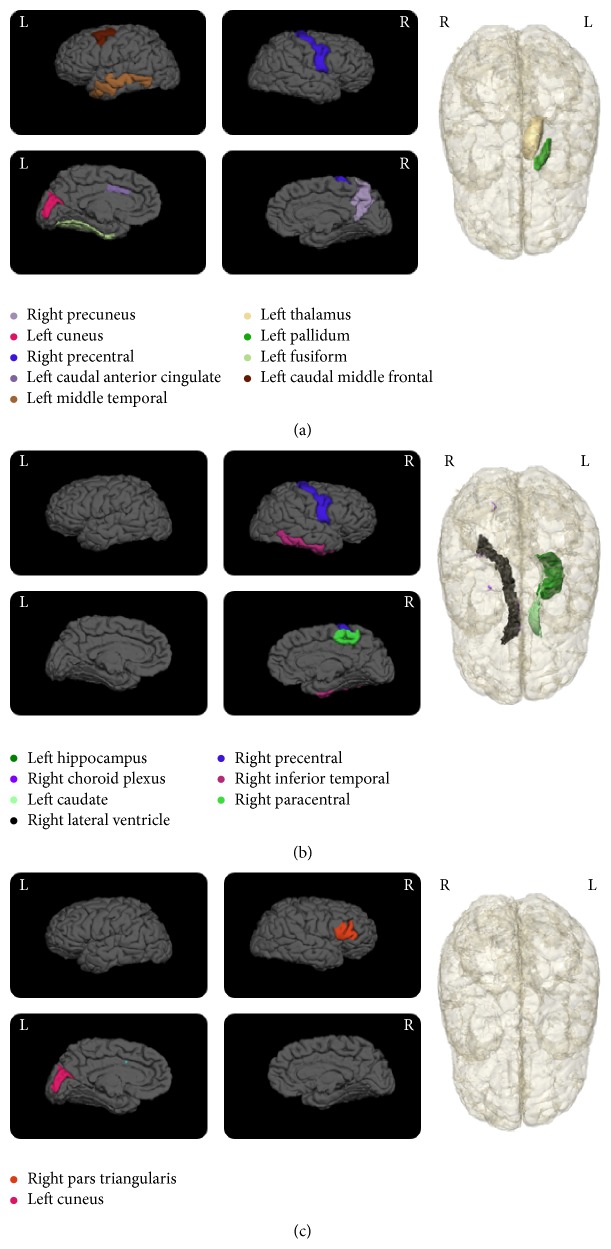
Cortical and subcortical maps showing the significant volumetric regions for each classification pair. (a) EMCI versus LMCI, (b) EMCI versus AD, and (c) LMCI versus AD. The regions are listed in Table 2. For each classification pair the letter “L” or “R” signifies the left and right hemisphere, respectively. Additionally, for the cortical representations, the top images in each set show the lateral view and the bottom images show the medial views.

The SLRM offer the opportunity to also rank these variables on the basis of their significance to each classification pair. The features listed in Table 2 are ranked according to the *P* value reflecting the significance of the variable towards the models. The ranking of the features displays the potential discriminating power of the different features in classification of the stages of AD. The anatomical distribution of the volumetric features, both cortical and subcortical, is displayed in Figures 3 and 4.

An important observation that can be made from Table 2 is the dominance of the neuropsychological parameters. It is seen in all the pairwise comparisons that the MMSE appears at very high ranks. MMSE is ranked as a significant feature in 4 of the 6 pairwise comparisons, whereas RAVLT shows up as significant in 2 of the 6 pairwise comparisons.

Interesting findings can also be seen in Figures 3 and 4 in terms of asymmetry in the distribution of the volumetric variables. For example, classification pairs such as CN versus EMCI and EMCI versus AD show dominance of the right hemisphere, where most of the significant feature volumes are from the right hemisphere. On the contrary, CN versus LMCI and EMCI versus LMCI show dominance of the left hemisphere. This asymmetrical distribution between groups can be a potential indicator of the shifts in atrophy patterns in the different stages of the disease. In the case of CN versus AD a more bihemispherical layout of the variables is observed as was reported in another study [34].

A closer inspection of the results shows that the top ranked volumetric variables, for example, hippocampus [35–37], ventricular [38, 39], cortical [35, 37, 40], and amygdala [39, 41], are all regions that have been proven to be effective predictors of AD and/or MCI by many other research groups. This observation is a strong indicator of the accuracy and usability of the ranking system developed in this study.

Also, cortical volumetric measures of the temporal, parietal, and cingulate regions show a marked presence in the significant groups. Other recent studies have also demonstrated the utility of regional temporal brain atrophy [38] and involvement of frontal lobe atrophy as important markers for AD staging [42].

### 3.2. Classification Performance

**Figure 5:**
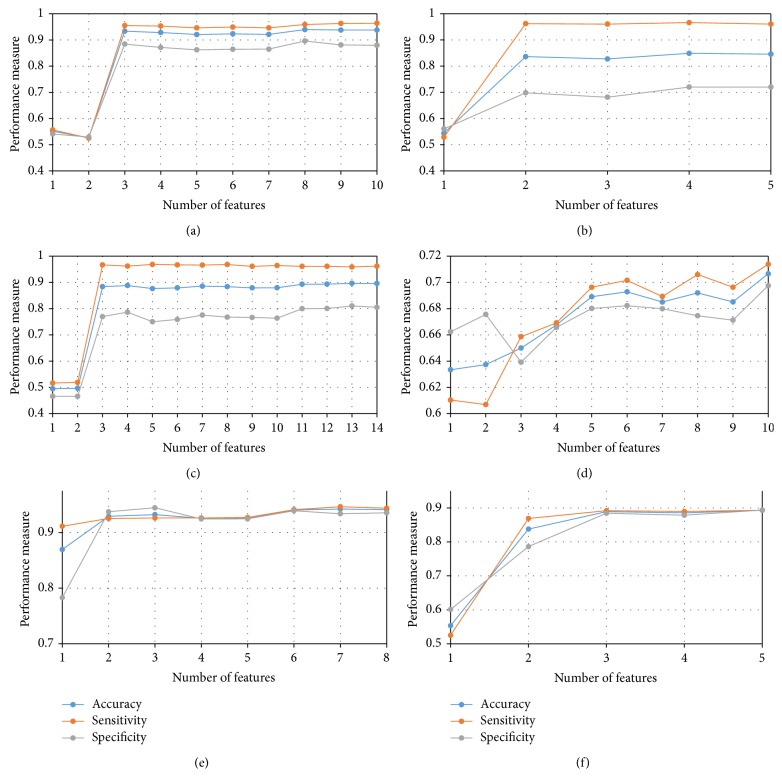
Results of incremental analysis displaying accuracy, sensitivity, and specificity as a function of the number of features used for the classifications. (a) CN versus AD, (b) CN versus EMCI, (c) CN versus LMCI, (d) EMCI versus LMCI, (e) EMCI versus AD, and (f) LMCI versus AD.

Figures 5(a)–5(f) show the classification accuracy, sensitivity, specificity, and precision for the 6 pairwise comparisons studies in this paper. The study performs an incremental analysis, whereby the classification is performed by adding an additional feature to the model starting with a single feature model. In other words, firstly only the top variable is used for classification and the performance is recorded. Following this the top 2 features are employed in the LDA classifier and so on and so forth. The results show a typical saturation effect whereby increasing the number of features in the classifier beyond a point does not improve the accuracy of the classifier.

Table 3 lists the highest accuracies obtained for each classification along with the number of features that were used to yield such accuracies. All results, displayed as Average ± Standard Deviation, indicate that, in all the cases except in the CN versus AD classification, the highest accuracy is indeed obtained when all the significant parameters, as enumerated in Table 2, are included in the LDA. In case of CN versus AD the first seven out of the ten features were able to define the optimal decisional space for the classification.

Table 3 also highlights the fact that the best classification accuracies are those obtained for groups, which are well separated diagnostically. For example, CN versus AD, CN versus LMCI, and EMCI versus AD show accuracies of 93.9%, 90.8%, and 94.5%, respectively. However, for groups which are not clearly differentiable like EMCI versus LMCI, an accuracy of 73.6% was achieved. Although this accuracy seems to be low, if not better, it is comparable to other studies found in literature, which have reported a similar accuracy in classification of MCI subjects [35, 43, 44].

Additionally, the choice of the prior probabilities used in the classification model can be derived from population's empirical estimates; that is, for the classification of AD subjects from CN the priors for the groups can be chosen to be 55/180 and 125/180 for the AD and CN groups, respectively. Although the choice of empirical prior probabilities may improve performance, the algorithm refrains from assigning empirical prior probabilities to reflect the nature of the problem in clinical environments, where distribution of subjects can be unknown.

### 3.3. Impact of Neuropsychological and Volumetric Features

Results shown in Figure 5 together with those given in Table 4 clearly illustrate the merits in combining neuropsychological measures with structural measures, where the combined model showed much improved accuracies for all the two-group classifications. For example, inclusion of the third ranked feature (MMSE) results in a sharp increase in accuracy in the CN versus AD classification.

In order to assess the merits of each category of these features, neuropsychological versus MRI measures, the classification algorithm was modified to operate separately using only either neuropsychological scores or MRI measures. For the neuropsychological model all neuropsychological features are used in the analysis, whereas in the MRI model only all the 115 MRI measures are used as features. In the combined model all neuropsychological and MRI measures are used concurrently. Please note that demographic variables, age, gender, and education, are used in all models as features to account for variability due to age and gender related changes often seen in Alzheimer's disease. Table 4 lists the average accuracy results obtained using a 2-fold LDA with 20 repetitions for classification of the different categories using only neuropsychological scores or MRI measures and then the combination of both.

It can be seen that the difference between the combined model and neuropsychological model alone is extremely small with the combined model offering a relative improvement of only 3.12% on average for the different classification pairs over a range of (0.33%–11.36%). However, it is interesting to note that the improvement offered by the combined model in case of EMCI versus LMCI, which are diagnostically very similar in cognition, is up to 11.36%. Additionally, MRI models alone offer on average accuracies of 71.2% (60%–84%). A large improvement in relative accuracy (24.59%) is thus obtained by combining neuropsychological scores with the MRI models.

Also, it is seen that for most of the classification pairs the Neuropsychological model offers a higher accuracy than the MRI model except for the EMCI versus LMCI classification pair. This finding shows that cognitive scores are not very good markers to differentiate between EMCI and LMCI and in such classification studies MRI based atrophy measures offer an added advantage.

### 3.4. Comparative Analysis

A comparison of classification performance with multiple studies that have appeared in literature is shown in Table 5, providing details of the respective imaging modality/biomarkers used in the study, the nature of the dataset used, and the classifier statistics in terms of the results obtained. The proposed approach achieved a very good performance relative to these studies. It is seen that for classification of AD subjects from CN the proposed technique fairs better than most of the studies. Another important point to note is that, except for Cuingnet et al. [36] and Zhou et al. [34], this study considered a larger database.

Moreover, most of the studies listed do not differentiate between the EMCI and LMCI groups but are pooled into a single larger MCI group. The availability of such data from the ADNI2 cohort made it possible for this study to explore this specific two-group classification and evaluate their distinctive features in the progression stages of AD.

The study by Klöppel et al. [37] shows the best results of all studies that only use MRI imaging based markers. For this study diagnosis of patients in Group I and Group II is confirmed using either histopathologically or neuropathologically with the aid of a biopsy or autopsy. Such a method of validation is extremely useful and most reliable for providing a more definite diagnosis that is less susceptible to errors caused by the subjectivity of the physician's diagnosis. Diagnosis in living subjects like those included in the proposed study naturally tends to be more subjective in nature. However, this does not hinder the intent or the merit of this study, which is to achieve high classification accuracy on a large population group with ease of applicability in a clinical setting.

Another important feature that can be observed from Table 5 is the comparative performance of the proposed study to multimodal imaging studies. It is shown that the classification performance achieved in this study is competitive and sometimes even better than other studies that relied on multiple imaging modalities. Although combination of modalities like PET and CSF provides valuable insight into the disease, their lack of availability across imaging centers and medical institutions hinder their potential integration in the decision making process in such facilities. A significant merit of the proposed technique is in its ability to achieve a very good classification performance using only MRI modality in conjunction with neuropsychological scores, all of which are routinely carried out for the diagnosis of AD.

The main limitation of this study was that the diagnosis of MCI and AD and the distinction of these two entities from normal aging are based on clinical measures, of which memory measures are paramount. On the other hand, structural MRI, as used in this study, measures volumetric changes in the brain. The severity of Alzheimer's pathology is only weakly correlated to cognitive and functional changes during life (in part because several other variables such as age and cognitive and brain reserve capacity can modify the correlations), but the pathology is strongly correlated to volumetric MRI changes. Furthermore, about 30−40% of cognitively normal elderly individuals have the pathology of Alzheimer's disease in their brains and these changes will be reflected as volumetric changes in their MRI scans, but not in their cognitive measures. Hence, using MRI measures, there is considerable overlap built in between “normal aging” and MCI/AD and so the classification between CN and MCI/AD is automatically at a disadvantage as compared to cognitive measures. It is, therefore, not surprising that we found MRI measures to provide only a small additional effect in separating CN from MCI/AD. A major advantage to using structural MRI scans is in distinguishing between different causes of dementia, such as separating the signature or atrophy patterns in Alzheimer's disease from frontotemporal dementia, vascular dementia and hydrocephalus, and distinguishing subtypes of Alzheimer's disease by the pattern of atrophy. These issues were not addressed as they were beyond the scope of this study, in which we addressed only the magnitude of contribution of volumetric MRI measures to the cognitive measures.

## 4. Conclusions

This study introduced a novel framework that combined MRI volumetric measures with neuropsychological scores in a statistically meaningful way. Consequently, a ranking of these features, structural and cognitive, proved very useful in constructing optimal decisional spaces for high-accuracy in two-group classifications. The highly ranked MRI measures proved effective in extracting the significant brain atrophy regions associated with AD and its prodromal stages for classifying cognitive normal subjects, EMCI, LMCI, and AD subjects. The feature extraction technique is based on a backward stepwise linear regression analysis, which demonstrated dominance of neuropsychological parameters like MMSE and RAVLT in delineating the different groups. The extracted features are also dominated by the presence of well-known subcortical atrophy regions like the hippocampus, amygdala, and ventricles and various temporoparietal and cingulate cortical regions. Classification results in two-group comparisons revealed a very high accuracy of 93.9%, 85.6%, 90.8%, 73.6%, 94.5%, and 90.1% for CN versus AD, CN versus EMCI, CN versus LMCI, EMCI versus LMCI, EMCI versus AD, and LMCI versus AD, respectively. The study also showed that a combination of MRI measures and neuropsychological parameters do yield better diagnostic results on average (accuracy: 87.6%) than using either MRI (accuracy: 71.2%) or cognitive scores (accuracy: 85.3%) alone. A practical merit of the proposed technique is in its ability to achieve high classification accuracy using only the MRI modality together with neuropsychological scores, all of which are routinely carried out for diagnosing AD.

## Figures and Tables

**Table 1:** Study data characteristics.

Characteristics	Cognitive normal (CN)	Early MCI (EMCI)	Late MCI (LMCI)	Alzheimer's Disease (AD)	*P* value^*∗*^
Number of subjects (*n*)	125	114	91	55	
Age (Years)	73.4 ± 5.9	70.7 ± 6.8	71.2 ± 7.8	75.4 ± 8.0	**<0.001**
Gender (male/female)	58/67	62/52	48/43	32/23	0.442^†^
Education (years)	16.4 ± 2.5	16.4 ± 2.7	16.6 ± 2.7	16.0 ± 2.4	0.720
CDRSb	0.0 ± 0.1	1.3 ± 0.9	1.9 ± 1.0	4.6 ± 1.6	**<0.001**
RAVLT	5.7 ± 2.4	5.7 ± 2.5	3.7 ± 2.4	2.0 ± 1.77	**<0.001**
MMSE	29.06 ± 1.17	28.58 ± 1.48	27.54 ± 1.82	22.80 ± 1.9	**<0.001**
TIV (mL)	1472.1 ± 148.3	1501.7 ± 148.8	1522.7 ± 163.6	1498.8 ± 169.7	0.123

Unless otherwise noted, data are presented as mean ± S.D.

^*∗*^ *P* value based on Student's *t*-test between CN and AD unless otherwise specified and those less than significance level 0.05 are bolded.

^†^A Fisher's exact test was performed, and *P* value shows that gender effect is not significant at significance level of 0.05.

**Table 2:** Significant features.

Rank	CN versus AD	*P* value	Rank	CN versus EMCI	*P* value
1	Left hippocampus volume	<0.001	1	Age	<0.01
2	Right lateral ventricle volume	<0.001	2	Right inferior temporal volume	<0.01
3	MMSE	<0.001	3	Left thalamus volume	<0.05
4	Right pars opercularis volume	<0.01	4	Right transverse temporal volume	<0.05
5	Age	<0.01	5	Right precuneus volume	<0.05
6	Education	<0.01			
7	Fourth ventricle volume	<0.05			
8	Left choroid plexus volume	<0.05			
9	Right precentral volume	<0.05			
10	Whole brain volume	<0.05			

Rank	CN versus LMCI	*P* value	Rank	EMCI versus LMCI	*P* value

1	Right hippocampus volume	<0.001	1	RAVLT	<0.001
2	Left superior parietal volume	<0.001	2	Right precuneus volume	<0.001
3	MMSE	<0.001	3	Left caudal anterior cingulate volume	<0.001
4	Age	<0.001	4	Left pallidum volume	<0.001
5	Left caudal anterior cingulate volume	<0.01	5	Left cuneus volume	<0.01
6	Left pericalcarine volume	<0.01	6	Left middle temporal volume	<0.01
7	Right superior parietal volume	<0.01	7	Left fusiform volume	<0.01
8	Left amygdala volume	<0.01	8	Left caudal middle frontal volume	<0.05
9	Right lateral occipital volume	<0.05	9	Right precentral volume	<0.05
10	Left caudate volume	<0.05	10	Left thalamus volume	<0.05
11	Left transverse temporal volume	<0.05			
12	Left posterior cingulate volume	<0.05			
13	Education	<0.05			
14	RAVLT	<0.05			

Rank	EMCI versus AD	*P* value	Rank	LMCI versus AD	*P* value

1	MMSE	<0.001	1	Age	<0.001
2	Left hippocampus volume	<0.001	2	MMSE	<0.001
3	Right lateral ventricle volume	<0.001	3	Non-WM hypointensities Volume	<0.01
4	Right inferior temporal volume	<0.001	4	Right pars triangularis volume	<0.05
5	Right choroid plexus volume	<0.001	5	Left cuneus volume	<0.05
6	Right precentral volume	<0.001			
7	Right paracentral volume	<0.01			
8	Left caudate volume	<0.05			

**Table 3:** Optimal accuracy obtained for each classification pair.

Classification	Number of features for maximum accuracy	Accuracy	Sensitivity	Specificity	Precision	*F* Measure
CN versus AD	7	0.939 ± 0.012	0.963 ± 0.021	0.895 ± 0.011	0.938 ± 0.026	0.948 ± 0.012
CN versus EMCI	5	0.856 ± 0.023	0.966 ± 0.012	0.741 ± 0.063	0.808 ± 0.062	0.871 ± 0.036
CN versus LMCI	14	0.908 ± 0.031	0.968 ± 0.024	0.837 ± 0.044	0.882 ± 0.054	0.919 ± 0.029
EMCI versus LMCI	10	0.736 ± 0.024	0.743 ± 0.055	0.727 ± 0.061	0.777 ± 0.082	0.758 ± 0.085
EMCI versus AD	8	0.945 ± 0.032	0.946 ± 0.022	0.950 ± 0.023	0.870 ± 0.016	0.951 ± 0.012
LMCI versus AD	5	0.901 ± 0.024	0.907 ± 0.044	0.893 ± 0.012	0.923 ± 0.033	0.914 ± 0.038

**Table 4:** Accuracy obtained for each classification pair for neuropsychological, MRI, and Combined Model.

Classification	Neuropsychological model	MRI measures model	Combined model	% Improvement offered by combined model over	
				Neuropsychological model^*∗*^	MRI measures model^†^
CN versus AD	0.922	0.842	0.939	1.844	11.520
CN versus EMCI	0.846	0.616	0.856	1.182	38.961
CN versus LMCI	0.885	0.714	0.908	2.599	27.171
EMCI versus LMCI	0.634	0.688	0.706	11.356	2.616
EMCI versus AD	0.932	0.814	0.945	1.395	16.093
LMCI versus AD	0.898	0.596	0.901	0.334	51.174

Average	0.853	0.712	0.876	3.118	24.589

^*∗*^% Improvement offered by combined model is calculated as (Combined Model/Neuropsychological Model)/Combined Model *∗* 100.

^†^% Improvement offered by combined model is calculated as (Combined Model/MRI Model)/Combined Model *∗* 1.

**Table 5:** Comparison of different methods.

#	Authors	Classification groups	Imaging modality/biomarkers	Source of data (Group 1/Group 2)	Repetition (cross validation)	Accuracy (%)	Sensitivity (%)	Specificity (%)
1	Zhang et al., 2011 [10]	CN, AD	MRI	ADNI (51/52)	10 (10-fold)	86.2	86.0	86.3
		CN, AD	CSF	ADNI (51/52)	10 (10-fold)	82.1	81.9	82.3
		CN, AD	PET	ADNI (51/52)	10 (10-fold)	86.5	86.3	86.6
		CN, AD	MRI, PET, CSF	ADNI (51/52)	10 (10-fold)	93.2	93.0	93.3

2	Hinrichs et al., 2011 [39]	CN, AD	MRI, PET	ADNI (48/66)	30 (10-fold)	87.6	78.9	93.8
		CN, AD	MRI, PET, CSF, APOE, cognitive scores	ADNI (48/66)	30 (10-fold)	92.4	86.7	96.6

3	Magnin et al., 2009 [40]	CN, AD	MRI	Private (16/22)	5000 (4-fold)	94.5	91.5	96.6

4	Klöppel et al., 2008 [37]	CN, AD	MRI	(Group I) Private (20/20)	LOOCV^‡^	95.0	95.0	95.0
		CN, AD	MRI	(Group II) Private (14/14)	LOOCV^‡^	92.9	100.0	85.7
		CN, AD	MRI	(Group III) Private (33/57)	LOOCV^‡^	81.1	60.6	93.0

5	Walhovd et al., 2010 [4]	CN, AD	MRI	ADNI (42/38)	N/A	82.5	N/A	N/A
		CN, AD	MRI, CSF	ADNI (42/38)	N/A	88.8	N/A	N/A

6	Cuingnet et al., 2011 [36]^*∗*^	CN, AD	MRI	ADNI (162/137)	N/A (2-fold)	N/A	82.0	89.0
		CN, AD	MRI	ADNI (162/137)	N/A (2-fold)	N/A	68.0	98.0

7	Zhou et al., 2014 [34]	CN, AD	MRI, MMSE	Private (129/60)	50 (2-fold)	92.3	88.2	94.2

8	Zhou et al., 2014 [41]	CN, AD	MRI, MMSE	Private (127/59)	50 (2-fold)	92.4	84.0	96.1
		CN, aMCI^††^	MRI, MMSE	Private (127/67)	50 (2-fold)	74.9	61.1	83.4
		CN, naMCI^††^	MRI, MMSE	Private (127/56)	50 (2-fold)	74.1	55.2	82.3

9	Desikan et al., 2009 [45]	CN, MCI^§^	MRI	OASIS^†^ (48/49)	N/A	84.0	73.0	94.0
		CN, MCI^§^	MRI	ADNI (57/94)	N/A	88.9	90.0	91.0

10	Davatzikos et al., 2008 [46]	CN, MCI^§^	MRI	BLAS^*∗∗*^ (15/15)	N/A	90.0	N/A	N/A

11	Fan et al., 2008 [47]	CN, AD	MRI, MMSE	ADNI (56/66)	LOOCV^‡^	94.3	N/A	N/A
		CN, MCI^§^	MRI, MMSE	ADNI (56/88)	LOOCV^‡^	81.8	N/A	N/A
		AD, MCI^§^	MRI, MMSE	ADNI (66/88)	LOOCV^‡^	74.3	N/A	N/A

12	Fan et al., 2008 [5]	CN, MCI^§^	MRI, PET	BLAS^*∗∗*^ (15/15)	LOOCV^‡^	90.0	N/A	N/A
		CN, MCI^§^	MRI	BLAS^*∗∗*^ (15/15)	LOOCV^‡^	87.0	N/A	N/A
		CN, MCI^§^	PET	BLAS^*∗∗*^ (15/15)	LOOCV^‡^	50.0	N/A	N/A

13	**Proposed Study**	CN, AD	MRI, cognitive scores	ADNI 2 (125/55)	20 (2-fold)	93.9	96.3	89.5
		CN, EMCI	MRI, cognitive scores	ADNI 2 (125/114)	20 (2-fold)	85.6	96.6	74.1
		CN, LMCI	MRI, cognitive scores	ADNI 2 (125/91)	20 (2-fold)	90.8	96.8	83.7

^*∗*^This paper by Cuingnet et al. [36] compares ten methods and results of methods with best sensitivity and specificity are shown here since accuracy is not reported.

^†^Oasis stands for Open Access Series of Imaging Studies (OASIS) database.

^§^The fraction Group 1/Group 2 lists the number of subjects in each classification group.

^‡^LOOCV: Leave one out cross validation.

^*∗∗*^BLAS: Baltimore Longitudinal Study of Aging.

^††^aMCI and naMCI represent amnestic and nonamnestic MCI, respectively.
